# Retrospective Evaluation of Pharmacist Interventions on Use of Antimicrobials Using a Clinical Surveillance Software in a Small Community Hospital

**DOI:** 10.3390/pharmacy4040032

**Published:** 2016-10-21

**Authors:** Samuel R. Huber, Fekadu Fullas, Kristel R. Nelson, Lesleigh B. Ailts, James M. Stratton, Michael T. Padomek

**Affiliations:** 1 Sentara Princess Ann Hospital, 2025 Green Mitchell Drive, Virginia Beach, VA 23456, USA; samrhuber@gmail.com; 2 UnityPoint Health-St. Luke’s, Pharmacy Department, 2720 Stone Park Boulevard, Sioux City, IA 51104, USA; kristel.nelson@unitypoint.org (K.R.N.); lesleigh.ailts@unitypoint.org (L.B.A.); james.stratton@unitypoint.org (J.M.S.); michael.padomek@unitypoint.org (M.T.P.)

**Keywords:** antibiotic interventions, antimicrobial stewardship program, clinical surveillance software, sentri7^®^

## Abstract

The Infectious Diseases Society of America and the Society for Healthcare Epidemiology of America “Guidelines for Developing an Institutional Program to Enhance Antimicrobial Stewardship” recommend the use of computer-based surveillance programs for efficient and thorough identification of potential interventions as part of an antimicrobial stewardship program (ASP). This retrospective study examined the benefit of utilizing a clinical surveillance software program to help guide antimicrobial therapy in an inpatient setting, in a small community hospital, without a formal ASP. The electronic health record (EHR) was used to retrieve documentations for the following types of antibiotic interventions: culture surveillance, duplicate therapy, duration of therapy and renal dose adjustments. The numbers of interventions made during the three-month periods before and after implementation of the clinical surveillance software were compared. Antibiotic related interventions aggregated to 144 and 270 in the pre- and post-implementation time frame, respectively (*p* < 0.0001). The total number of antibiotic interventions overall and interventions in three of the four sub-categories increased significantly from the pre-implementation to post-implementation period. Clinical surveillance software is a valuable tool to assist pharmacists in evaluating antimicrobial therapy.

## 1. Introduction

Antimicrobial stewardship has been gaining attention as infections caused by multidrug resistant organisms and costs associated with antibiotic use escalate across the country. Highlighting the need for intervention, the Centers for Medicare and Medicaid Services (CMS) issued a finalized version of an infection control survey in 2015 that included written policies and procedures to improve antibiotic use [1]. The Infectious Diseases Society of America (IDSA) has published two different guidelines on antimicrobial stewardship [2,3]. Both guidelines recommend the use of clinical decision-support and surveillance programs. Current studies that focus on the use of surveillance programs have been conducted at academic medical centers with an established ASPs [4,5]. These studies have shown decreased antibiotic costs [4] and an upsurge of interventions [4,5]. In a study that covered antibiotic use over a seven-year period, Pestotnik et al. [6] demonstrated a decrease in antibiotic cost and antibiotic-associated adverse drug events with the use of antibiotic management support programs. Evans et al. [7] showed similar trends in antibiotic use, where implementation of a computer-assisted management program significantly improved antibiotic-susceptibility mismatch, adverse reactions, dose adjustment and a reduction in cost of antibiotics and length of hospital stays. Various software programs can be linked to EHRs, such as Epic (Verona, WI, USA), and Cerner (North Kansas City, MO, USA), to streamline the ASP. Algorithms are configured in the software programs by end users [8]. The system allows hospitals to customize clinical rules for appropriate antibiotic use in a given setting. 

The use of clinical surveillance software, such as Sentri7^®^ (Bellevue, Washington, DC, USA), reduces the time needed for interventions as compared to reviewing various daily paper reports [9]. By interfacing with EHRs, the tools allow pharmacists to evaluate antibiotics, culture results and clinical diagnoses [10,11]. Sentri7^®^, which is linked to Epic at UnityPoint Health-St. Luke’s, was reviewed daily by clinical pharmacists. The diverse categories populate in Sentri7^®^ when potential opportunities for changes in therapies are possible. Pharmacists use promptings from Sentri7^®^ to direct a more thorough evaluation of each patient regarding specific areas of concern. The purpose of this study is to evaluate the impact of a clinical surveillance software Sentri7^®^ on the number of interventions made by pharmacists at a community hospital without an established ASP.

## 2. Methods

The study was approved by the institutional review board. It was conducted at UnityPoint Health-St. Luke’s, a 151-bed community hospital in Sioux City, IA, USA, which does not have a formal ASP. It was a retrospective evaluation of all electronically documented pharmacist interventions for three months before (May to July, 2014) and after (September to November, 2014) implementation of a clinical surveillance software (Sentri7^®^, Wolters Kluwer Health). The month of implementation was excluded to allow pharmacy staff familiarization with the program. Prior to the institution’s use of the clinical surveillance software, pharmacists did not have the same quality and quantity of opportunities for interventions. Regarding antibiotics, information was found most often when health care providers consulted pharmacy staff for input, when pharmacists noticed potential issues on reviewing patients’ charts for other problems, or based on culture reports sent from the laboratory. Interventions were recorded through an Epic *i*-vent function in the course of routine order reviews and verifications by different pharmacists. There was no systematic way of identifying antimicrobial stewardship interventions. After implementation, pharmacists on clinical shifts were tasked with reviewing Sentri7^®^ through the course of the day and acting on all alerts. Through the use of Sentri7^®^, review of patients’ charts was streamlined. 

The EHR was used to identify all interventions documented by pharmacists in the specified time periods. Interventions were included in the analysis if they were able to be placed in one of the following sub-categories: culture surveillance, antibiotic renal dose adjustment, duration of antibiotic therapy, or duplicate antibiotic therapy. Interventions were excluded from the analysis if they were physician-initiated (including pharmacy-to-dose consults) or if there was insufficient documentation to correctly categorize the intervention. If multiple interventions were recorded under the same documentation, each documented intervention was counted only once.

The total numbers of interventions and those interventions within each antibiotic sub-category in the pre- and post-implementation periods were compared for statistical significance by using the chi-square (χ^2^) test. The total number of patient days in the respective two periods was used to compare the rates of interventions. 

## 3. Results

A total of 3576 interventions were reviewed in the pre-implementation and 4409 in the post-implementation period. Out of this, 144 and 270 interventions in the respective periods were antibiotic-related. The average admission rate was 881 per month in both periods, while the total patient days were 9397 and 10,217 for the three-month period before and after implementation of the program, respectively, caused by slightly longer hospital stays. In the pre-implementation period, 10 antibiotic interventions were excluded for lack of information and 89 were excluded due to being physician-initiated. In the post-implementation period, 19 antibiotic interventions were excluded for lack of information and 144 were excluded due to being physician-initiated. 

Each sub-category of antibiotic interventions was analyzed (Table 1). The most common intervention type in both periods was antibiotic dose adjustment, followed by culture surveillance (Table 1). A total of 144 interventions in the pre-implementation and 270 in the post-implementation period were compared. There were 1.6 and 2.6 interventions per 1000 patient days, respectively in the two periods (*p* < 0.0001). Significantly more interventions were made post-implementation in the culture surveillance, duration of antibiotic therapy, and duplicate antibiotic therapy categories. The difference in the number of interventions in the antibiotic renal dose adjustment category was not significant. As a result of antibiotic interventions, 45 out of 144 (31.3%) and 98 out of 270 (36.3%) recommendations were accepted during pre- and post-Sentri7^®^ periods, respectively.

## 4. Discussion

Sentri7^®^ provides intervention suggestions after an alert is generated. The software supports antimicrobial management by triggering alerts from as many as 150 pre-built rules. It also provides an antibiotic review based on laboratory culture and susceptibility reports [9]. Before implementation of the Sentri7^®^ software program at our institution, clinical pharmacists received a facsimile of all positive cultures from the laboratory each day, although it was not consistently reviewed. This proved to be a time consuming and cumbersome process. There were no other set methods of reviewing for potential antibiotic related interventions. Prior to implementation, the clinical rules for the software were developed by a team of clinical pharmacists at UnityPoint Health and then customized locally by our institution at the time of the implementation of the program (Table 2). 

Alerts for interventions were produced immediately in Sentri7^®^ software, which prompted clinical pharmacists to review all alerts in the program on a daily basis and make appropriate interventions, either by leaving notes for physicians in the EHR, directly contacting physicians, or making changes as allowed by the hospital formulary and collaborative practice guidelines.

While studies have shown the benefit of the use of a clinical surveillance software, there is a lack of data on its use in hospitals without an established ASP and dedicated infectious disease pharmacists. This study showed that implementation of a clinical surveillance software in a hospital without an established ASP can increase the number of antibiotic interventions. Interventions increased in multiple categories related to antimicrobial stewardship, showing that this can be a valuable tool to support clinical pharmacist generalists. When antimicrobial stewardship programs become a requirement for all hospitals, incorporation of clinical decision support tools, such as Sentri7^®^, will be helpful.

As site-specific rules were developed at our institution, the challenges encountered were related to the cost of purchasing Sentri7^®^ software, validations to ensure clinical data were crossing correctly from main EHR (Epic) to Sentri7^®^ and testing the rule sets. The pharmacy staff was in-serviced before roll-out. Since the software rules were intuitive and easy to follow, it did not take long for the staff to be acclimated to the ASP.

There are two potential explanations for the lack of significant differences in the dose change category. One is the exclusion of pharmacy-to-dose consults in the review. Many renal dose changes would have been included under such consults, and the tool likely helped pharmacists catch necessary dose changes after the initial consult. A second explanation is that renal dosing was already being successfully captured upon initial verification before use of the tool. Further analysis is warranted to describe the impact of such effects.

## 5. Limitations

One limitation of this study is the timing of the post-implementation period. This time period was chosen in order to limit the analysis to the original rules, as new rules were regularly added to the program in the year after implementation. Because the analysis immediately followed implementation, pharmacists were acclimating to the program. Increases in interventions would be expected as new rules are added, pharmacists become more comfortable with use of the tool, and physicians get accustomed to different pharmacy recommendations. Also, interventions were only counted if proper documentation is done in the EHR, and some interventions may not have been documented.

A second limitation of this study is the use of a number of interventions as the primary outcome. While increased pharmacist interventions may be assumed to lead to benefits in direct patient care, variables such as length of hospitalization and possible antimicrobial cost savings, further analysis would be needed to determine such correlation.

Generalizability of these results may also be limited due to the lack of formal antimicrobial stewardship activities prior to implementation. Hospitals that already have robust ASPs or experienced infectious disease pharmacists may not see a significant increase in the number of interventions made.

## 6. Conclusions

The total number of relevant antibiotic interventions increased significantly from pre-implementation to post-implementation of Sentri7^®^. Significant increases were seen in all categories except for antibiotic dose adjustment. Based on its ability to help pharmacists more consistently and efficiently identify interventions, clinical surveillance software is a valuable tool in smaller hospitals to assist pharmacists in evaluating antimicrobial therapy. Going forward, conditions of participation will require all hospitals to develop and implement programs to improve antibiotic use.

## Figures and Tables

**Table 1:** Comparison of antibiotic interventions before and after implementation of Sentri7^®^.

	Pre-Implementation Period	Post-Implementation Period	
	Total Number of Interventions	
	(*n* = 144)	(*n* = 270)	*p* #
**Type of Antibiotic Interventions**			
Culture surveillance	11	67	<0.0001
Renal Dose adjustment	128	168	NS *
Duration of therapy	3	15	0.0156
Duplicate therapy	2	20	0.0006

# Adjusted for average number of patient days per month for each period. * NS = not significant.

**Table 2:** Categories of clinical rules in the surveillance program for intervention *.

Rule Category	Individual Rules/Medications Include
Culture Surveillance (Drug-Bug Mismatch)	*Clostridium difficile* positive without oral coverage
	Cefazolin with resistant organism
	*Candida albicans* on echinocandins
	Quinolone with resistant organism
	Penicillin-sensitive *Enterococcus faecalis* on vancomycin/linezolid
	Carbapenem with resistant organism
	ESBL positive organism without carbapenem
	Cefepime and cefepime-resistant organism
	Piperacillin/tazobactam and piperacillin/tazobactam-resistant organism
	Methicillin-sensitive Staphylococcus aureus on vancomycin/daptomycin/linezolid/tigecycline
	Ertapenem with *Pseudomonas*
Antibiotic use surveillance #	
Duration of Therapy	Antimicrobial use >72 h and no signs of infection (T > 38.3 °C, WBC > 12, ANC > 7.5, or Bands > 10%)
	Antimicrobial use >7 days
Duplicate Therapy	Anaerobic double coverage
	Concomitant cefepime and levofloxacin >2 days
	Duplicate beta-lactam use
	Concomitant piperacillin/tazobactam and levofloxacin >2 days
Renal dosing adjustments	Acyclovir, amoxicillin, ampicillin, ampicillin/sulbactam, aztreonam, cefaclor, cefazolin, cefdinir, cefepime, cefotaxime, cefoxitin, ceftazidime, cefuroxime, cephalexin, ciprofloxacin, daptomycin, ertapenem, famciclovir, fluconazole, levofloxacin, meropenem, metronidazole, nitrofurantoin, oseltamivir, piperacillin/tazobactam, sulfamethoxazole/trimethoprim, valacyclovir

* ANC = Absolute neutrophil count in (×10^3^ in cubic millimeter); ESBL = Extended-spectrum beta-lactamase; T = temperature in degrees Celsius; WBC = White blood cell count (×10^9^ per L). # Antibiotic use >72 h with no sign of infection and without re-evaluation; antibiotic use >7 days for active infection without re-evaluation; double-coverage and concomitant antibiotic use >2 days without assessment for step-down therapy.
